# Treatment provider is most predictive of ED dismissal in minimally-injured trauma patients: a retrospective review

**DOI:** 10.1186/1752-2897-7-5

**Published:** 2013-05-16

**Authors:** Diane L S Hunt, Gina M Berg, Rosalee E Zackula, Francie H Ekengren, Diana Lippoldt, Elizabeth Ablah, Ruth Wetta

**Affiliations:** 1 Wesley Medical Center Trauma Services, Wichita, Kansas; 2 Kansas Surgical Consultants, Wichita, Kansas; 3 University of Kansas School of Medicine – Wichita, Preventive Medicine and Public Health, Wichita, Kansas; 4 Medical Staff Office, Wesley Medical Center, 550 N. Hillside, Wichita, KS 67214, Kansas

**Keywords:** Critical care, Emergency medicine, Hospitalizations, Physicians, Trauma centers, Triage

## Abstract

**Background:**

Secondary triage protocols have been described in the literature as physiologic (first-tier) criteria and mechanism-related (second-tier) criteria to determine the level of trauma activation. There is debate as to the efficiency of triage decisions based on mechanism of injury which may result in overtriage and overuse of limited trauma resources. Our institution developed and implemented an advanced three-tier trauma alert system in which stable patients presenting with blunt traumatic mechanism of injury would be evaluated by the emergency department (ED) physician rather than the trauma surgeon. The American College of Surgeons Committee on Trauma (ACSCOT) requires that operational changes be monitored and evaluated for patient safety and performance. The primary aim of this study was to evaluate the process, as well as outcomes, of patient care pre and post implementation of the new triage protocol. The secondary aim was to determine predictor variables that were associated with ED dismissal.

**Methods:**

A retrospective blinded pre/post process change implementation explicit chart review was conducted to compare process and outcomes of minimally injured trauma patients who were field triaged by mechanism of injury. Generalized linear modeling was performed to determine which predictor variables were associated with ED dismissal.

**Results:**

There were no significant differences in minutes to physician evaluation, CT scan, OR/ICU disposition, readmission rates, safety or quality. Significant differences only occurred in time to chest x-ray, length of stay in ED, and ED dismissal rates. Trauma surgeon and ED physician patient groups did not differ on ISS, age, or sex. The only significant predictor for ED dismissal was treatment provider, with ED physicians 3.6 times more likely to dismiss the patient from the emergency department.

**Conclusions:**

ED physicians provided compble care as measured by safety, timeliness, and quality in minimally-injured patients triaged to our trauma center based only on mechanism of injury. Moreover, ED physicians were more likely to dismiss patients from the ED. A three-tiered internal triaging protocol can redirect resource usage to reduce the burden on the trauma service. This may be increasingly beneficial in trauma models in which the trauma surgeons also serve as critical care intensivists.

## 

Trauma systems were originally developed to quickly identify and prioritize patients with significant injuries and transport them to trauma centers for surgical evaluation and treatment [1]. The Committee on Trauma Field Triage Decision Scheme in the American College of Surgeons (ACSCOT) optimal resource guide [1-3] is a triage system based on mechanism of injury as well as the physiologic and anatomic pmeters of the patient during the evaluation. While the Field Triage Decision Scheme is a tool for emergency responders to determine transportation to the appropriate level of care within the trauma system, trauma centers use the common data points (physiologic, anatomic, mechanism of injury criteria) as a starting point for developing internal triage schematics to determine appropriate trauma activation at the bedside. Some trauma systems have devised and incorporated secondary triage protocols with two-tier alerts [4-8]. These may be defined as physiologic (first-tier) criteria and mechanism-related (second-tier) criteria to determine the level of trauma activation [1,3,9].

There is debate as to the efficiency of triage decisions based on mechanism of injury in the otherwise physically stable trauma patient. The sensitivity of triage decision-making is increased which often results in decreased specificity, or overtriage [1,10]. While the ACSCOT (2006) allows for a generous buffer of overtriage (25%-50%) to keep the rate of undertriage to an acceptable rate of 5%, it is suggested that secondary triage is necessary to improve the accuracy of the triage process, especially in disaster management. The need for full trauma team activation in stable blunt (as indicated by mechanism of injury (Step Three, ACS Field Triage Decision Scheme) trauma patients [11-13] and pediatric trauma patients [9] has been questioned in the literature. Green (2006) [14], after his review of the literature, concluded that there is a lack of objective evidentiary basis to support the premise that routine surgeon presence is necessary for optimal trauma outcomes. Moreover, he calls for further research to determine which trauma patients require surgical presence on the initial phase of resuscitation.

Redirecting trauma resuscitation for minimally-injured patients to evaluation by an emergency department (ED) physician is time and resource efficient [3]. Our institution, a Level I Trauma Center located in the Midwest, has experienced an increase in the number of trauma patients of over 400% since its first ACS verification in 1991. Recorded volumes in 2002 and 2003 for trauma were 1953 and 1899 respectively and 54,749 and 57,991 for the ED. In response to greater demands placed on the trauma system and in an effort to better utilize existing resources and maintain quality of medical care, we developed and instituted an advanced, three-tiered triage trauma system with a new category of trauma patients, Tier III Alerts. The new three-tiered trauma alerts decision scheme changed the classification of patients suffering a blunt (non-penetrating) injury (as indicated by mechanism of injury according to Step Three Field Triage Decision Scheme) to be seen by an ED physician trained in Advanced Trauma Life Support. Patients could not have abnormalities in physiologic or anatomic pmeters (Tier I with full trauma team activation), nor chest or abdominal complaints or a witnessed loss of consciousness of greater than five minutes or change in mentation (Tier II with partial trauma team activation). The radiology, nursing, and respiratory therapist trauma support staff did not change as a result of the Tier III trauma activation. If, after post-implementation of the new triage protocol, a patient required immediate surgical intervention by a trauma surgeon, the patient trauma alert was revised to a Tier I or Tier II by the ED physician. Moreover, a trauma surgeon evaluated all patients not dismissed from the ED. This protocol was developed under the review of this institution’s trauma services and implemented during an American College of Surgeons (ACS) Level I Trauma Center review period.

The American College of Surgeons Committee on Trauma (ACSCOT) mandates that trauma programs should have a goal to provide efficacious and evidence based care, with operational processes that promote patient safety while being cost effective [2]. Further, it is expected that performance and quality will be measured and evaluated. The ACSCOT divides trauma system performance into two types of measurement: 1) process measures and 2) outcome measures. Examples of process measures, which may be defined by institutional and/or evidence based guidelines, as related to this study include but are not limited to: appropriateness of emergency department triage, and delays in assessment, diagnosis or treatment. Outcome measures include, but are not limited to: hospital admission and readmission.

## Goals

The purpose of this study was to determine if a change from a two-tier to a three-tier trauma response system affected patient safety and care. A further aim was to identify predictors of ED dismissal of minimally-injured trauma patients. Process measures and outcomes of patients who met trauma activation criteria based on mechanism of injury [2] were examined before and after implementation of the internal triage decision scheme.

## Methods

### Study design and population

This was a retrospective blinded explicit (trauma nurse) pre/post process change implementation chart review conducted to compare process and outcomes of minimally injured trauma patients who were field triaged by mechanism of injury. All minimally injured trauma patients were included in the study if they met trauma criteria by Step Three (mechanism of injury) in the Field Triage Decision Scheme [2]. Patients were excluded if they experienced: loss of consciousness > 5 minutes, abdominal and/or chest pain, long bone and/or open fracture, pregnancy, obvious intoxication/substance abuse and/or Glasgow Coma Scale was ≤ 13. Falls from standing and isolated hip fractures were also excluded. In the pre-implementation (control group November 2001 to March 2002), patients were evaluated immediately at bedside in a trauma bay by trauma surgeon or resident (same day evaluation by attending surgeon). In the post-implementation (treatment group November 2002 to March 2003), patients were evaluated at bedside in a trauma bay by the emergency department (ED) physician. This study received institutional review board approval by Wichita Medical Research and Education Foundation.

### Measurements

Data were gathered from the trauma registry and patient medical records. Patient information included: age, sex, mechanism of injury (e.g. motor vehicle crash, pedestrian injury, fall), method of arrival, admission Glasgow Coma Scale (GCS), and Injury Severity Score (ISS). Treatment process measures included: time to initial physician evaluations (patient arrival to physician at bedside as recorded by trauma control nurse), time to chest x-ray (patient arrival to procedure performed), time to CT scan (patient arrival to procedure performed), and inclusion in trauma registry. Treatment outcomes included ED length of stay, patient disposition, and returns to the ED within 30 days.

### Data analysis

The results are presented in means and confidence intervals or frequencies and percentages as appropriate. T-tests or Chi-square analysis were used to evaluate the similarity and appropriateness of sub-group comparison. Mean comparison of independent variables was analyzed using an independent samples t-test. Chi-square tests or Fisher’s exact test was used to analyze frequency data. Factors associated with outcome (dismissed/not dismissed) were analyzed using the binomial probability distribution in generalized linear models. A variety of link functions were explored [15]. Criteria for model selection included two Goodness of Fit measures: Deviance and Akaike’s Information Criterion (AIC). Data were analyzed using the PASW Statistics (SPSS) Version 18.0 [16].

## Results

Two hundred forty-seven patient records were identified; four records were excluded due to incomplete data, thus, 243 charts were included in this study. Demographic, clinical characteristics, process and outcome data of the study population are described in Table 1. The study sample was half male (52%) and had a mean age of 33.9 years. The median, (range and interquartile range (IQR)) for GCS [17], and ISS [18] were 15 (Range 14–15; IQR 15–15) and 4.5 (Range 1–18; IQR 2–5) respectively. ISS median (range and IQR) for patients evaluated by trauma surgeons and ED physicians were 5.0 (Range 1–17; IQR= 4–6) and 4.0 (Range 1–18; IQR= 1–5) respectively. Table 1 demonstrates the categorization (frequency and percentage) of GCS (14 or 15) and ISS (1–4 or 5–18; split at median) as used in the regression model. Comparisons of demographic and clinical characteristics are also listed in Table 1 and revealed no statistical differences in the comparison groups based on number of sample size, age, sex, and mechanism of injury, GCS or ISS distribution.

**Table 1:** Patient demographics, clinical characteristics and outcomes

	**Trauma population**	**Treatment provider**		
**Variables**	**Total sample**	**Trauma surgeon (Control)**	**ED physician (Intervention)**	p*
*Number (%)*	243	113 (47)	130 (53)	.28
*Patient demographics*				
?Mean age (CI)	33.9 (31.1 - 36.6)	31.9 (28.3 - 35.5)	35.5 (31.4 - 39.6)	.20
?Sex: Males (%)	126 (51.9)	54 (47.8)	72 (55.4)	.29
?Injury: MVC/Accident (%)	137 (56.4)	69 (61.1)	68 (52.3)	.27
?Injury: Fall (%)	55 (22.6)	25 (22.1)	30 (23.1)	--
?Injury: Other (%)	51(21.0)	19 (16.8)	32 (24.6)	--
*Clinical characteristics (n=232)*				
?GCS^‡^=14 (%)	38 (15.8)	20 (17.9)	18 (14.0)	.41^†^
?GCS^‡^=15 (%)	203 (84.2)	92 (82.1)	111 (86.0)	
?ISS^§^ =1-4 (%)	116 (50.0)	49 (46.7)	67 (52.8)	.36^†^
?ISS^§^ =5-18 (%)	116 (50.0)	56 (53.3)	60 (47.2)	
*Minutes to care by*				
?Physician evaluation	1.9 (1.0 - 2.7)	2.1 (1.0 - 3.1)	1.7 (.33 - 3.1)	.66
?Chest X-ray (n=150)	11.8 (9.1 - 14.5)	9.2 (7.4 - 10.9) (n=89)	15.7 (9.6 - 21.8) (n=61)	**.02**
?Head CT (n=127)	13.2 (9.6 - 16.7)	10.7 (8.0 - 12.3) (n=63)	16.1 (9.4 - 22.8) (n=64)	.10
*ED length of stay (minutes)*	102 (96–109)	93 (84–102)	111 (102–121)	**.006**
*Disposition*				
?Discharged (%)	99 (40.7)	26 (23.2)	72 (55.8)	**<.001**
?Admitted (%)	144 (59.3)	86 (76.8)	57 (44.2)	**--**
*Readmission rates (%)*	3 (1)	2 (2)	1 (<1)	.48

* independent sample t-test; frequency comparison by Pearson's Chi-square; CI=confidence interval; CT=computed tomography.

GCS and ISS reported by grouping as entered into regression model; ISS groups determined by median split.

†Mann–Whitney U test;

^‡^GCS (Glasgow Coma Scale): [17].

^§^ISS (Injury Severity Score): [18].

### Process measures: time to care and in care

Comparisons of outcomes are listed at the bottom of Table 1. There was no difference between the trauma surgeons and ED physicians in patients’ time to initial evaluation or time to head CT scan. There was a significant difference in patients’ time to chest x-ray (ED physicians mean time was 15.7 minutes versus trauma surgeons’ mean time of 9.2 minutes).

The patients’ ED length of stay was statistically different. Patients treated by trauma surgeons spent a mean time of 93 minutes in the ED, whereas patients treated by ED physicians spent a mean time of 111 minutes in the ED.

### Outcomes: patient disposition

The patient disposition (dismissal/non-dismissal) is listed by provider in Table 1. ED physicians dismissed a significantly greater proportion of patients (56%) than did trauma surgeons (23%).

### Quality assurance (QA)

There was no difference in the QA of trauma patients, as all trauma patients were entered into the trauma registry [2,19]. There was not a significant (p=.48) difference in readmission (same facility) rates (Table 1) between trauma surgeons (2%) and ED physicians (<1%). Table 2 provides characteristics of the three patients who were readmitted within 30 days of discharge or dismissal. There were seven (3%) patients (Table 3) who were retrospectively assigned an ISS > 15 [20]. Of those six were admitted to the hospital. Five of those were initially evaluated by the ED physician and elevated to a Tier II for surgical evaluation. No patients who had ISS >15 were readmitted to the hospital (same facility within 30 days).

**Table 2:** Characteristics of the patients who were readmitted with 30 days

**Patient**	**Provider**	**Age**	**ISS**	**GCS**	**Original disposition**
237	Trauma surgeon	26	5	14	Admitted to floor
28	Trauma surgeon	38	5	15	Admitted to floor
31	ED physician	31	1	15	Dismissed to home

Note: ISS = Injury Severity Score; GCS=Glasgow Coma Scale.

**Table 3:** Patients who had ISS score >15

**Patient**	**Provider**	**Age**	**ISS**	**GCS**	**Original disposition**
176	ED physician	*	18	15	Admitted to SICU
227	ED physician	66	17	14	Admitted to floor
219	ED physician	74	17	14	Dismissed to home
210	ED physician	72	17	15	Admitted to floor
187	ED physician	*	17	14	Admitted to floor
65	Trauma surgeon	68	17	15	Admitted to SICU
180	ED physician	22	16	14	Admitted to SICU

Note: ISS = Injury Severity Score;*> age 80.

### Generalized linear model

A generalized linear model was conducted to determine whether the predictor variables – age, sex, ISS, GCS, treatment provider were associated with patient outcome (dismissed/not dismissed) of minimally-injured trauma patients. Of the original 243 cases, 11 were dropped from the analysis due to missing data on at least one of the variables leaving 232 cases, (137 not dismissed and 95 dismissed). The final model included the following variables (Table 4): age (pediatric 0–18; adult 19–54; older adult 55+), GCS (14 or 15); ISS (as determined by median split range 1–4 or range 5–18); and treatment provider (trauma surgeon or ED physician); Deviance = 1.049 and Akaike’s Information Criterion (AIC) = 67.978. Treatment provider was the only significant predictor in the model; patients treated by ED physicians were dismissed from the ED significantly more often than patients treated by trauma surgeons (p<.001). Controlling for all other variables in the model, the relative risk indicated that ED physicians were 3.6 times more likely to dismiss than trauma surgeons for this minimally-injured trauma population.

**Table 4:** Relationship between predictor variables and patient disposition

					**95% CI**	
		Wald (df=1)	*p*	RR*	Lower	Upper
Provider						
	ED physician	26.865	< 0.001	3.641	2.234	5.935
	Trauma surgeon			ref		
GCS						
	14	1.794	0.180	0.588	0.27	1.279
	15			ref		
ISS						
	Scores = 5 to18	2.803	0.094	0.693	0.451	1.065
	Scores = 1 to 4			ref		
Age groups						
	Pediatric (0–18)	1.536	0.215	1.34	0.844	2.129
	Older adult (55+)	1.745	0.187	0.636	0.325	1.244
	Adults (19–54)			ref		

Note: Generalized Linear Model, binomial distribution, complementary log-log link function; ref=reference group.

GCS=Glasgow Coma Scale; ISS=Injury Severity Score; p=probability; df=degrees of freedom; RR=Relative Risk; CI=confidence interval.

Additional tests with variables entered into the model as interactions revealed no significant associations, thus were not reported.

## Discussion

These results demonstrate that minimally-injured patients admitted to the trauma system based on mechanism of injury alone receive the same timeliness and quality of care if seen by an ED physician rather than a trauma surgeon and is congruent with other literature [3,13]. Further, ED physicians identified and transferred care to the trauma surgeon when appropriate. As a result, the triage modification promoted more effective use of trauma surgeons’ time by permitting patients with less severe injuries to be evaluated and treated by ED physicians. This may have increased importance in trauma models that include in-house trauma surgeons who also serve as critical care intensivists.

Seven (3%) patients were assigned (post-treatment) an ISS score > 15; which indicates undertriage, but within the suggested acceptable undertriage rate of five percent (ACS 2006). Of those, six (post-implementation intervention group) were initially evaluated by the ED physician and upgraded appropriately to the trauma surgeon for hospital admission.

Although ED physician patients experienced a longer ED length of stay and greater wait for CT scan, this does not indicate patient safety was compromised. Variation in wait time may be due to the availability of ED physicians as they see all ED patients whereas trauma surgeons only evaluate patients identified as trauma. The additional time a patient spends in the ED also may have been used for observation rather than admitting for overnight observation.

The difference between trauma surgeons and ED physicians in the proportion of ED dismissals (which may be due to patient injuries and co-morbidities) suggests a bias in dismissal practices representing differences in decision-making processes. Differences in practitioner training may lead to the variation in responses to minimally-injured trauma patients, in that trauma surgeons may be more likely to admit the patient and observe whereas ED physicians are trained to respond with a “treat and release” perspective. Another factor in the higher dismissal rate for ED physicians may be the need to request trauma surgeon involvement if the patient is not dismissed. While treatment provider was the only significant predictor of dismissal, with ED physicians dismissing 3.6 times more often than trauma surgeons, it should be noted that when appropriate the trauma patients initially evaluated by ED physicians in the Tier-Three Model were additionally evaluated by the trauma surgeon.

This expands upon earlier findings suggesting that ED physicians are appropriate care providers (noting that patients can be upgraded to trauma team activation if necessary) for minimally-injured trauma patients [3,13,19]. This will reduce the need for routine surgeon presence on trauma patient arrival [4] as well as findings that less medical staff (trauma team) is needed for the stable, minimally injured patient [21,22]. This modification in triage rules from two-tiered to three-tiered trauma alerts allows for less involvement by the trauma surgeon in caring for stable trauma patients triaged by mechanism of injury criteria, keeping in mind triage needs for special populations such as the elderly [11,23,24], pregnant [2], and pediatrics [9]. Furthermore, the modified triage rules appears to reduce the burden of care on the trauma surgeon, unnecessary patient admissions and may be extrapolated into improved cost-effectiveness which warrants further investigation to confirm. Evaluation of changes in operational processes for performance improvement and quality assurance is necessary to ensure optimal outcomes.

### Limitations

Changes in trauma and ED staff as well as practice patterns, may have changed between the study periods. The retrospective design may be subject to differing documentation of the initial patient presentation; however, this challenge was addressed by employing the same triage nurse to identify charts for both the pre-process change (control) and post-process change (treatment) groups, as well as using the same time of year to help control for seasonal changes in method of injury. Statistical similarity of the groups in age, sex, GCS at admission, ISS, and method of injury, validate the comparisons.

## Conclusions

This investigation, evaluating acceptable physician practices as well as patient outcomes before and after the operational transition from a two-tier trauma alert model to three-tier trauma alert model, found that ED physicians provided compble care as measured by safety, timeliness, and quality in minimally-injured patients triaged to our trauma center based on mechanism of injury only. A three-tiered internal triaging schematic, either internally developed or refined from the Field Triage Decision Scheme as defined by the American College of Surgeons, can redirect resource usage to reduce the burden on the trauma service. This may be increasingly beneficial in trauma models in which the trauma surgeons also serve as critical care intensivists. Moreover, ED physicians were more likely to dismiss patients from the ED which warrants further investigation for cost effectiveness. Any operational changes in trauma care to promote should be evaluated for both process and quality outcomes to ensure patient safety. The operational transition to the three-tiered triage model, as described here, met both process and quality criteria and further has been approved by the American College of Surgeons (ACS) within a Level I Trauma Center reverification review.

## Abbreviations

ACSCOT: American College of Surgeons Committee on Trauma; ACS: American College of Surgeons; AIC: Akaike’s information criterion; CI: Confidence interval; CT scan: Computed tomography; df: Degrees of freedom; ED: Emergency department; GCS: Glasgow coma scale; ICU: Intensive care unit; IRB: Institutional review board; ISS: Injury severity score; MVC: Motor vehicle collision; OR: Operating Room; p: Probability; QA: Quality assurance; ref: Reference group; RR: Relative risk

## Competing interests

The authors declare they have no competing interests.

## Authors’ contributions

DLSH: developed the original research question and manuscript development. GMB: conception, statistical analysis and manuscript development. REZ: statistical analysis and manuscript development. DL: conception and manuscript development. FHE: manuscript development and critical revisions. EA: conception and manuscript development. RW: conception and manuscript development. All authors read and approved the final manuscript.
